# Séroprévalence des marqueurs viraux sur les dons du sang au Centre de Transfusion Sanguine, Hôpital Militaire d’Instruction Mohammed V de Rabat

**DOI:** 10.11604/pamj.2016.25.185.6266

**Published:** 2016-11-24

**Authors:** Jean Uwingabiye, Hafidi Zahid, Loubet Unyendje, Rachid Hadef

**Affiliations:** 1 Centre de Transfusion Sanguine, Hôpital Militaire d’Instruction Mohammed V de Rabat, Faculté de Médecine et de Pharmacie de Rabat, Université Mohammed V, Rabat, Maroc

**Keywords:** Séroprévalence, VHB, VHC, VIH, don de sang, Seroprevalence, HBV, HCV, HIV, blood donation

## Abstract

Le but de ce travail était de déterminer la prévalence du virus de l’immunodéficience humaine (VIH), du virus de l’hépatite B (VHB) et C (VHC) sur les dons du sang collectés au Centre de transfusion sanguine(CTS) de l’hôpital militaire d’instruction Mohammed V entre 2010 et 2012. Etude rétrospective menée auprès des donneurs de sang militaires âgés de 18 à 50 ans avec prédominance masculine (95%). L’entretien médical pré-don constitue la première barrière de sélection des sujets à risque. Le dépistage biologique était réalisé par technique immuno-enzymatique en milieu liquide utilisant des anticorps et/ou des antigènes. L’ELISA (enzyme linked immuno-sorbent assay) combiné de quatrième génération pour VHC et VIH a été utilisé. La confirmation a été faite en réalisant la même technique en double au CTS et au laboratoire de virologie. Dans notre série de 25661 échantillons testés, la prévalence du VHB était 3,97‰ (n=102), celle de VHC était 2,45 ‰ (n=63), celle de VIH était 0,15 ‰ (n=4). Un seul cas de coïnfection (0,039 ‰) par le VHB et VHC a été noté, aucune association entre VIH-VHB, VIH-VHC ou VHB, VHC et VIH n’a été enregistrée. Les taux faibles de séroprévalence des marqueurs viraux de notre étude montrent l’amélioration des mesures préventives en ce qui concerne la sélection des donneurs et des tests de dépistage. Cette prévalence constatée incite à maintenir l’utilisation du réactif combiné qui est la seule alternative à la biologie moléculaire pour les pays en voie de développement.

## Abstract (en)

This study aims to determine the prevalence of human immunodeficiency virus (HIV) infections and hepatitis B virus (HBV) and hepatitis C virus (HCV) infections among blood donors at the Blood Donor Center, Mohammed V Military Teaching Hospital between 2010 and 2012. We conducted a retrospective study among military blood donors aged 18-50 years, with a male predominance (95%). Pre-donation interview is the first selection barrier for individuals at risk. Biological screening was performed by liquid enzyme immunoassay technique using antibodies and/or antigen. Fourth generation combined HCV and HIV antigen/antibody ELISA (enzyme-linked immunosorbent assay) test was used. The Blood Donor Center and the laboratory of virology used the same technique performed in duplicate to confirm results. Out of 25661 tested samples, the prevalence rate of HBV infections was 3.97 ‰ (n = 102), the prevalence rate of HCV infections was 2.45 ‰ (n = 63) and the prevalence rate of HIV infections was 0.15 ‰ (n = 4). A single case with HBV and HCV virus co-infection (0.039 ‰) was registered, no association between HIV-HBV, HIV-HCV or HBV, HCV and HIV infections was recorded. The low seroprevalence rates of viral markers recorded in our study show improvement in preventive measures for donor selection and screening tests. The registered prevalence encourages the use of combined reagent, which is the only alternative to molecular biology in developing countries.

## Introduction

Au cours de vingt dernières années, la sécurité en transfusion sanguine a fait de très grand progrès vis-vis du risque infectieux et immunologique. Selon la législation marocaine de transfusion sanguine, le dépistage du virus de l’immunodéficience humaine (VIH), du virus de l’hépatite B (VHB) et C (VHC), de la syphilis et le dosage des Alanine Amino-Transaminases (ALAT) font partie des examens obligatoires sur le sang objet du don. Les infections dues au VIH, VHB et VHC sont des problèmes majeurs de santé publique à travers le monde [1–4]. Les personnes porteuses du VHB et/ou du VHC sont exposées aux risques de passage à la chronicité avec la survenue de complications tel que la cirrhose et le carcinome hépatocellulaire. Selon les estimations de l’Organisation mondiale de la santé(OMS), environ 2 milliards de personnes vivent avec le VHB dont plus de 240 millions sont porteurs d’une hépatite B chronique et entre 500 000 et 700 000 personnes meurent chaque année en raison de l´infection par le VHB [2–5]. Près de170 millions de personnes sont porteurs chroniques du VHC et plus de 350 000 personnes meurent chaque année de maladies hépatiques liées au VHC [2–5]. A l’échelle mondiale, 33,4 millions de personnes sont infectées par le VIH [1]. La surveillance épidémiologique de ces infections chez les donneurs de sang permet de suivre la prévalence et de repérer les principaux moyens de lutte et de prévention de leur dissémination par la transfusion. Le but du présent travail est d’évaluer les séroprévalences des marqueurs viraux chez les donneurs militaires et les comparer avec autres populations afin de faire le point sur l’épidémiologie locale.

## Méthodes

Il s’agit d’une étude rétrospective menée auprès des donneurs militaires âgés de 18 à 50 ans avec prédominance masculine (95%) au Centre de transfusion Sanguine(CTS) de l’Hôpital militaire d’Instruction Mohammed V(HMIMV), Rabat(Maroc), réalisée sur une période de trois ans allant du 1er janvier 2010 au 31 décembre 2012. L’entretien médical pré-don a été faite pour sélectionner les sujets à risque.

Le dépistage biologique était réalisé par une technique immuno-enzymatique basée sur le principe de la technique en sandwich en milieu liquide utilisant les anticorps et/ou les antigènes à l’aide de l’automate Evolis. L’ELISA (enzyme linked immuno-sorbent assay) combiné dit de 4ème génération pour le VIH et VHC a été utilisée. La recherche des marqueurs du VIH a été effectué par le test GenscreenTM ULTRA VIH Ag-Ab (Bio-rad, France) qui permet la détection de l´antigène p24 du VIH et des divers anticorps associés au virus HIV-1 et/ou HIV-2. La détection de l´antigène du virus de l´hépatite B de surface (Ag HBs) est effectuée à l’aide du test MonolisaTM Ag HBs ULTRA (Bio-rad, France) et pour le sérodiagnostic de l’infection de l’hépatite C, MonolisaTM HCV Ag-Ab a été utilisé pour la détection de l´antigène de capside et des anticorps Anti-HCV (Bio-rad, France).

Les tests sérologiques négatifs ont été considérés négatifs pour les infections dépistées. Tout don dont le test ELISA positif a été écarté du don et la confirmation a été faite en réalisant la même technique en double au CTS et au laboratoire virologie de l’HMIMV en utilisant le même réactif ou un réactif différent de celui de dépistage. La discordance entre les deux tests ou la répétabilité du test positif entraine l’élimination du don et la prise en charge des sujets concernés.

## Résultats

Entre 2010 et 2012, sur 25661 échantillons testés, 102 dons ont été confirmés positifs pour le VHB (3,97 ‰), ce taux était environ 2 fois plus élevé que celui du VHC avec une séroprévalence de 2,45 ‰ (63 dons) et 15 fois plus élevé que celui du VIH avec une séroprévalence de 0,15 ‰ (4 cas). La coïnfection entre VHB et VHC a été détectée dans un seul cas, soit 0,039 ‰. Aucune association entre le VIH-VHB, VIH-VHC ou VHB, VHC et VIH n’a été enregistrée (Tableau 1).

**Tableau 1:** Séroprévalence de VHB, VHC et VIH durant la période de 2010-2012

	2010	2011	2012	Total
Nombre total des dons	8245	8232	9184	25661
Nombre des dons VHB positifs	35	37	30	102
La prévalence du VHB	4,2	4,5	3,3	3,97
Nombre des dons VHC positifs	29	17	17	63
La prévalence du VHC	3,5	2	1,8	2,45
Nombre des dons VIH positifs	2	0	2	4
La prévalence du VIH	0,24	0	0,22	0,15

## Discussion

La spécificité des caractéristiques sociodémographiques de la population faisant l’objet de cette étude concerne notre échantillon qui est essentiellement composé par les donneurs militaires adultes à prédominance masculine, jeunes, bénévoles et réguliers. Le CTS de l’HMIMV s’occupe de la disponibilité des donneurs en collectant les produits sanguins et en assurant la fidélisation des donneurs et une meilleure sécurité transfusionnelle.

**Figure 1:**
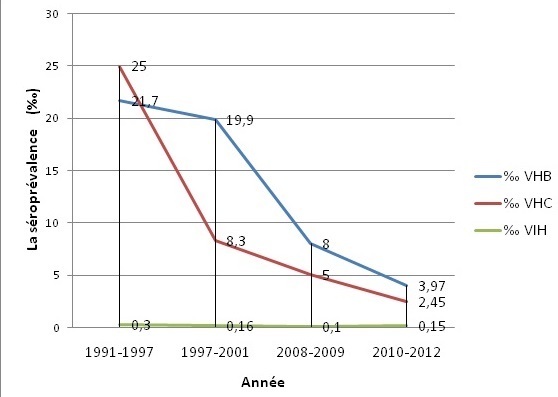
Évolution de la prévalence des marqueurs viraux au Centre de Transfusion Sanguine de l’hôpital militaire d’instruction mohammed V (1991-2012)

En comparaison des études effectuées dans la même institution [6–8] (Tableau 2) (Figure 1), la prévalence du VHB a baissé depuis 1991 et on a aussi observé une baisse de la prévalence du VHC sur la période 1991-2012. Alors que le taux de prévalence du VIH a diminué sur la période de 1991-2009, ce taux a sensiblement augmenté entre 2010 et 2012 [6–8]. Par comparaison, le taux de prévalence du VHB observé dans notre étude est 4 fois plus faible que celui de la population générale marocaine (16,6‰) [9], celui du VHC est environ 8 fois plus faible que celui observé dans la population générale marocaine (19,3‰) [10] et la séroprévalence de VIH dans la population générale marocaine (1,4‰) [11] est 9 fois plus élevé que celle obtenue dans notre étude. Ces données témoignent l’efficacité de la sélection des donneurs du sang au CTS de l’HMIMV.

**Tableau 2:** L’évolution de la séroprévalence des marqueurs viraux durant la période de 1991-2012

Marqueurs viraux	1991-1997[6]	1997-2001[7]	2008-2009[8]	2010-2012
‰ VHB	21,7	19,9	8	3,97
‰ VHC	25	8,3	5	2,45
‰ VIH	0,3	0,16	0,1	0,15

Les séroprévalences chez les donneurs du sang du Maghreb sont proches de ceux de notre étude sauf la Lybie qui présente une forte prévalence du VHC et VIH [12] et une étude réalisée en Tunisie a montré aussi une prévalence élevée de l’hépatite B [13] (Tableau 3). Les taux de notre étude sont nettement supérieurs à ceux observés chez les donneurs du sang en France et aux Etats-Unis d’Amérique [14, 15] (Tableau 3) soulignant l´importance des efforts à fournir en ce qui concerne la sensibilisation et l’information de la population marocaine et en l’occurrence la population militaire. Les taux obtenus sont nettement inférieurs à ceux observés chez les donneurs de sang des pays de l’Afrique sub-saharienne [16, 17] (Tableau 3). La faible prévalence des marqueurs du VHB et VHC témoigne que le Maroc est une zone d’endémicité intermédiaire tandis que l’Afrique sub-saharienne est une zone de haute endémicité avec une séroprévalence élevée.

**Tableau 3:** Séroprévalence des marqueurs viraux chez les donneurs du sang

Auteurs	Pays	‰ VHB	‰ VHC	‰ VIH
Khmmaj A et al. 2010 [12]	Libye	12-24	85-189	0-29
Safer(2006) [13]	Tunisie	5O-100	4-18	-
Pillonel J et al.2012 [14]	France	0,73	0,38	0,037
Zou S et al.2012 [15]	Etats-Unis d’Amérique	0,078	0,334	0,028
Ymele FF et al. 2012[16]	Cameroun	121,4	14,4	44,4
Tessema B et al. 2010 [17]	Ethiopie	47	7	38
Notre étude	Maroc	3,97	2,45	0,15

En ce qui concerne les coïnfections, elles sont plus rares; la présence simultanée de VHB et VHC est détectée dans un seul cas (0,039‰) et ces résultats sont beaucoup plus bas que ceux obtenus dans les pays d’Afrique sub-saharienne [16, 17]. Dans une étude rapportée au Cameroun, le taux prévalence des cas de coïnfections entre VIH et le VHB était de 0,78%, celle de VHB-VHC était 0,22%, 0,06% pour le VIH –VHC et la coïnfection entre les trois virus était 0,04% chez les donneurs de sang [16]. Les coïnfections du VHB, VHC et VIH sont graves et fréquent en Afrique sub-saharienne [16]. En effet, ces virus possèdent le même mode de transmission : des rapports sexuels non protégés, la consommation des drogues par voies intraveineuses, transmission de la mère à l´enfant et la transfusion sanguine et les greffes d’organes. En outre, leur coïnfection a des effets plus négatifs. L’association de l’infection du VIH, VHB et du VHC est caractérisée par une plus fréquente évolution vers la chronicité, une augmentation de taux de réplication virale, une réactivation virale conduisant à une augmentation de la progression vers la fibrose et la cirrhose du foie [18, 19]. Les mesures préventives de ces infections consistent à appliquer strictement le respect des règles d’hygiène universelles; le lavage des mains, les mesures de désinfection et de stérilisation habituelles, ainsi que le recours aux matériels à usage unique, l’éducation sexuelle et la vaccination contre le VHB.

## Conclusion

Les taux faibles de séroprévalence des marqueurs viraux de notre étude montrent l’amélioration des mesures préventives en ce qui concerne la sélection des donneurs et des tests de dépistage surtout l’utilisation de réactifs combinés qui représente le meilleur alternatif à la biologie moléculaire dans les pays en voie de développement. Pour maintenir une faible prévalence, il faut surveiller l’évolution de ces marqueurs et promouvoir des actions de sensibilisation et d'information pour obtenir l’auto-exclusion totale.

### Etat des connaissances actuelles sur le sujet

- Les infections dues au virus de l’immunodéficience humaine (VIH), virus de l’hépatite B (VHB) et C (VHC), sont des problèmes majeurs de santé publique à travers le monde;
- La surveillance épidémiologique des infections due au VIH, VHB et VHC chez les donneurs de sang permet de suivre leurs prévalences et de repérer les principaux moyens de lutte et de prévention de leur dissémination par la transfusion.

### Contribution de notre étude à la connaissance

- Cette étude montre que la séroprévalence des marqueurs viraux chez les donneurs du sang militaires au Maroc est inférieure à celle observé chez les donneurs de sang des pays de l’Afrique sub-saharienne et elle est aussi supérieure à celle observé chez les donneurs du sang en France et aux Etats-Unis;
- Les taux de prévalence des marqueurs viraux obtenus dans notre étude sont nettement inferieurs à ceux de la population générale marocaine ce qui témoigne l’efficacité de sélection des donneurs du sang dans notre institution.
